# Pharmacological Studies in Eating Disorders: A Historical Review

**DOI:** 10.3390/nu16050594

**Published:** 2024-02-22

**Authors:** Yael D. Lewis, Lukas Bergner, Holger Steinberg, Jessica Bentley, Hubertus Himmerich

**Affiliations:** 1 Hadarim Eating Disorders Unit, Shalvata Mental Health Centre, Hod Hasharon 4534708, Israel; 2 Faculty of Medicine, Tel Aviv University, Tel Aviv 6997801, Israel; 3 Forschungsstelle für die Geschichte der Psychiatrie, Klinik und Poliklinik Psychiatrie und Psychotherapie, Medizinische Fakultät der Universität Leipzig, 04103 Leipzig, Germany; lukas.bergner@gmx.de (L.B.); holger.steinberg@medizin.uni-leipzig.de (H.S.); 4 Department of Psychological Medicine, Institute of Psychiatry, Psychology & Neuroscience, King’s College London, London SE5 8AF, UK; jessica.bentley@kcl.ac.uk (J.B.); hubertus.himmerich@kcl.ac.uk (H.H.); 5 South London and Maudsley NHS Foundation Trust, London BR3 3BX, UK

**Keywords:** psychopharmacology, history, eating disorders, medication

## Abstract

Eating disorders (EDs) are serious mental health conditions characterised by impaired eating behaviours and nutrition as well as disturbed body image, entailing considerable mortality and morbidity. Psychopharmacological medication is an important component in the treatment of EDs. In this review, we performed a historic analysis of pharmacotherapeutic research in EDs based on the scientific studies included in the recently published World Federation of Societies for Biological Psychiatry (WFSBP) guidelines for ED treatment. This analysis focuses on early approaches and trends in the methods of clinical pharmacological research in EDs, for example, the sample sizes of randomised controlled trials (RCTs). We found the development of psychopharmacological treatments for EDs followed advancements in psychiatric pharmacotherapy. However, the application of RCTs to the study of pharmacotherapy for EDs may be an impediment as limited participant numbers and inadequate research funding impede generalisability and statistical power. Moreover, current medication usage often deviates from guideline recommendations. In conclusion, the RCT model may not effectively capture the complexities of ED treatment, and funding limitations hinder research activity. Novel genetically/biologically based treatments are warranted. A more comprehensive understanding of EDs and individualised approaches should guide research and drug development for improved treatment outcomes.

## 1. Introduction

Eating disorders (EDs) are defined by a persistent disturbance in eating and body image, which affects food consumption and leads to significant impairment of physical health or psychosocial functioning [1]. EDs are widespread, with a recent systematic review demonstrating a rise in their prevalence from 3.5% in studies conducted between 2000 and 2006 to 7.8% in those conducted between 2013 and 2018 [2]. EDs are serious psychiatric conditions with considerable morbidity [3] and mortality [4,5]. Indeed, the standardised mortality rate in patients with anorexia nervosa (AN) is five times higher than that of healthy controls [6], with suicide being a major cause of death [7]. With the prevalence of EDs continuously rising [2], they impose a growing cost on health services. Estimates suggest that 20 million people in the European Union have an ED, with financial and burden-of-disease costs of approximately EUR 1 trillion per year [8]. Guidelines for the treatment of EDs are mainly based on psychological interventions [9,10]; however, a substantial proportion of patients still experience a protracted illness course and poor prognosis [11]. This reinforced the need for other treatment modalities, including pharmacotherapy. The strong genetic–biological basis for EDs held promise for pharmacotherapeutic targets in the nervous system as well as the metabolic and immune systems [12].

This review aims to examine pharmacotherapy for EDs from a historical perspective. Following an initial summary of early approaches to medication use in the treatment of EDs, a chronological analysis of clinical research for drug therapy was performed, with a specific focus on the rationale and effects of the agents used. Trends in research and their implications were also analysed. We based our narrative summaries and figures on the studies reviewed in the recently published update of the World Federation of Societies for Biological Psychiatry (WFSBP) guidelines for the pharmacological treatment of EDs [13]. For the 2023 WFSBP ED guidelines, an individual systematic review was performed for each ED using the medical database PubMed, including studies from 2011, when the previous WFSBP guidelines were published [14] until the end of 2021. Search terms were extracted from the chapter on Feeding and Eating Disorders of DSM-5 [1] and previous reviews or meta-analyses on pharmacology for EDs. The search was supplemented by Internet searches, hand searches of reference lists of included articles and related reviews. Articles were included if they described studies testing a pharmacological treatment in the respective ED targeting core ED symptoms, e.g., weight, restriction, binge eating episodes, and purging. For inclusion, the studies had to be performed using a randomised controlled trial (RCT) design; however, they were still included if there were no existing RCTs for the specific treatment, e.g., open trials or case reports. Reported measurable outcomes were also necessary for inclusion. Exclusion criteria included no application of pharmacological treatment, no measurable outcomes, a reported outcome that did not include core ED symptoms, articles that were not original publications (e.g., meeting abstracts), articles reporting animal studies or articles not written in English [13]. Studies from the previous 2011 WFSBP guidelines [14] were re-evaluated for inclusion also in the latest guidelines according to the criteria above. Data loading, cleaning and analysis were performed using R version 4.2.2. The effects of drugs were determined from the published articles and classified as weight gain for anorexia nervosa (AN), binge reduction and purge reduction for bulimia nervosa (BN), and binge reduction and weight loss for binge eating disorder (BED). Likewise, funding information was gathered from the published articles, with any pharmaceutical involvement classified as pharma funding and any specific mention of an academic grant classified as academic funding. Plots were created using the ggplot2 package [15]. All studies per disorder were included in timeline analyses. Only placebo-controlled studies were included in the effects analysis and funding analysis. This included 96 RCTs, 19 crossover trials, 3 open trials with placebo arms and 1 retrospective case–control study.

## 2. Restrictive Eating Disorders and Anorexia Nervosa

Therapeutic concepts for the treatment of food refusal and psychiatric underweight patients developed from the beginning of the 19th century. These included force-feeding via stomach tubes, special nutritional regimens or specific behavioural methods that could be considered psychotherapy and psychoeducation in a broader sense today. In these methods, the therapists’ role model function and encouragement were of key importance. Pharmacological treatments do not appear to have played a major role in the 19th century, although some evidence can be found. For example, in around 1850, individual case histories were published in the psychiatric literature reporting that chloroform (trichloromethane), initially used as an anaesthetic primarily in obstetrics and surgery, was being used for the treatment of psychiatric illnesses and syndromes, specifically for psychotic food refusal [16].

A study that analysed 18 important German-language psychiatric school textbooks from 1803 until today found early pharmacotherapeutic approaches to underweight or food refusal [17]. One of the first to address this was Heinrich Neumann, a prominent 19th-century psychiatrist who was the director of the private asylum for the insane in Pöpelwitz, Silesia (part of Poland today), and later director of the Psychiatric University Hospital in Breslau. In his ‘*Lehrbuch der Psychiatrie*’ (*Textbook of Psychiatry*), published in 1859, he recommended iodine potassium for the treatment of anorexic patients, as it ‘‘seems to be highly effective’’ in inducing appetite. In addition to iodine potassium, Neumann [18] discussed the application of tartar emetic, or potassium antimonyl tartrate, which he suggested produced a much longer appetite-increasing effect. In this context, it is noteworthy that Johann Christian August Heinroth, appointed as the world’s first professor of psychiatry in Leipzig in 1811, suggested tartar emetic as an effective therapy for melancholia [19].

In the second half of the 19th century, the English physician Sir William Withey Gull and the French Neurologist Ernest-Charles Lasègue described specific cases of anorexia for the first time in medicine almost simultaneously by comprehensively describing their symptoms [20]. Gull also termed the condition ‘anorexia nervosa’ in his ground-breaking work, depicted in the 1873 paper ‘Anorexia Nervosa (Apepsia Hysterica, Anorexia Hysterica)’. For its treatment, he did not only recommend “some form of nourishing food every two hours, as milk, cream, soup, eggs, fish, chicken”, but stated, “with the nourishment, I would conjoin a dessert-spoonful of brandy every two or three hours” [21]. The precise dose and frequency of brandy can be seen as the first pharmacological treatment recommendation for underweight patients with AN.

In 1941, August Bostroem recommended using insulin to increase appetite [22]. Insulin was used in small doses to avoid hypoglycaemia in all patients refusing food intake [16,23,24]. Similar recommendations for insulin were already given by Lange in the first edition of his textbook in 1935 [25].

William Sargant practised psychiatry at St Thomas’ Hospital in London between the 1930s and 1960s. He started his career in an era when physical therapies, including insulin, electroconvulsive therapy and psychosurgery, were gaining prominence over psychotherapy, an important step in establishing the place of psychiatry as a medical profession [26]. In 1960, a novel method for the treatment of AN was presented in a landmark paper that included large oral doses of chlorpromazine and modified insulin infusions [27]. The serendipitous discovery of chlorpromazine and its notable effects in alleviating anxiety and agitation in psychiatric patients led to a transformation in the treatment of mental illnesses, with the relative emptying of psychiatric asylums with sales and pharmaceutical financial success [28]. For AN, the rationale was that insulin and chlorpromazine would induce appetite among starved patients and reduce anxiety due to chlorpromazine’s effect as a “major tranquiliser” [29].

The pharmacological methods mentioned are based on motivating appetite by means of medication. The psychiatrists of the 19th and earlier 20th centuries gave their advice on the basis of their own extensive clinical experience and the findings of colleagues. This reflects the clinical science as it was understood in their time, i.e., expert opinion based on clinical experience, which later transformed into case–control studies and open trials. It was not until the 1960s and 1970s that RCTs attained dominance in clinical pharmacotherapy research. The American Food and Drug Administration (FDA) came to require proof of safety and efficacy, and RCTs became the standard for proving both. Focus turned to population-based research, measurable outcomes and replicable interventions, ultimately challenging the dominance of psychoanalysis and reinforcing the importance of drug treatments and disease specificity [28].

As clinical trials did not reveal positive evidence for the then-accessible therapies, they were quickly abandoned, and the newly discovered, evidence-based antipsychotics and antidepressants gained prominence. This led to a swing in treatment approaches, with growing recognition of the importance of persuasion and reassurance to enable re-nourishment and cure, with a key role in psychotherapy [29].

**Figure 1:**
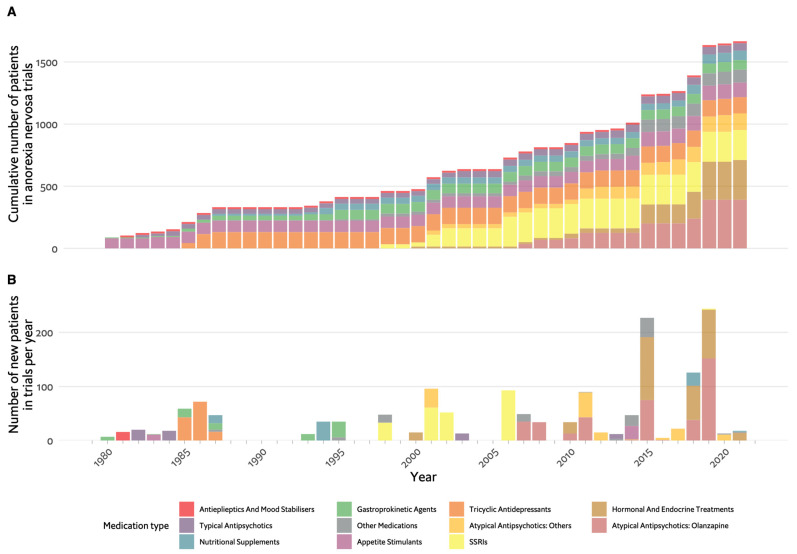
Timeline of pharmacological studies in anorexia nervosa by medication group. (**A**). Cumulative number of patients who participated in studies, colour coded by medication group. (**B**). Number of new participants per year in studies, colour coded by medication group. SSRIs: selective serotonin reuptake inhibitors.

Although the pendulum for AN placed emphasis on psychotherapy and behavioural treatment methods [10], their inadequate outcomes left room and motivation for further exploration of potential pharmacological treatment. Figure 1 illustrates progress in the clinical research of modern-day pharmacotherapy for AN in the past 50 years. While the timeline included case studies, most of the evidence comes from studies already conducted as open controlled trials or RCTs in the framework of evidence-based medicine (EBM). As seen in Figure 1A, the cumulative number of participants in pharmacological clinical studies has not yet reached 2000. Figure 1B demonstrated that rarely has the yearly number of new participants in published trials exceeded 100 has mostly been considerably lower. In comparison, an extensive systematic review and network meta-analysis of antidepressants for the acute treatment of major depressive disorder included data from ~500 RCTs with over 100,000 participants [30], and a similar network meta-analysis on the acute treatment of psychosis in multi-episode schizophrenia included ~50,000 participants in ~400 RCTs [31].

**Figure 2:**
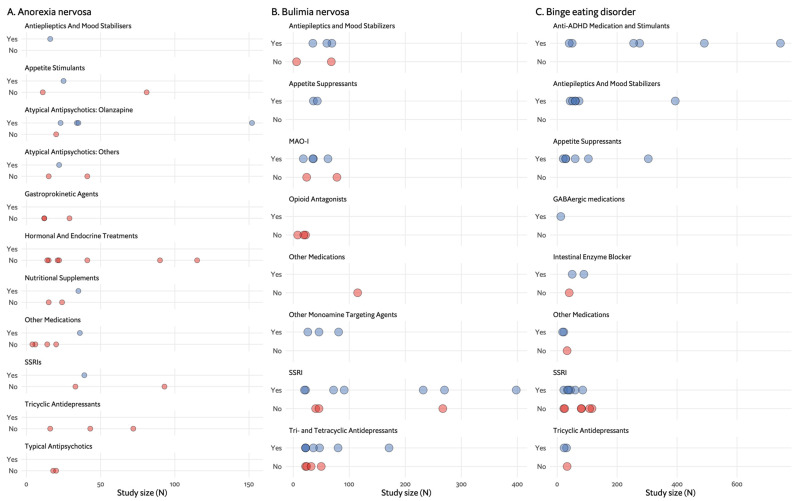
Effects found in placebo-controlled trials by medication group and number of participants (N). Each dot represents a single study. (**A**). Effect (weight gain) found in anorexia nervosa placebo-controlled trials. (**B**). Effects (binge and/or purge reduction) found in bulimia nervosa placebo-controlled trials. (**C**). Effects (binge reduction and weight loss) found in binge eating disorder placebo- controlled trials.

As shown on the timeline in Figure 1, the drugs tested parallel the major advances in psychopharmacology in this period: investigation of typical antipsychotics and tricyclic antidepressants (TCAs) in the 1980s, selective serotonin reuptake inhibitors (SSRIs) in the 2000s and atypical antipsychotics up until recently. However, appetite stimulants and hormonal and endocrine treatments were investigated throughout the entire period between 1980 and 2023. Figure 2A demonstrates whether the tested drug led to weight gain in placebo-controlled trials, which is the main outcome for most studies in AN. The majority of studies included fewer than 50 participants and had negative results. Olanzapine was the only drug for which there are several studies with positive findings, with one of these being the largest RCT in AN, having included 152 randomised participants [32].

One of the first classes of drugs to be evaluated was the neuroleptic or typical antipsychotics, a direct continuation of the pharmaceutical revolution in psychiatry since the 1950s. Clinical trials on pimozide and sulpiride for AN were conducted due to a proposed hyperactive dopaminergic cerebral system mechanism implicated in AN and following some successful case studies [33,34]. There were 38 participants in these placebo-controlled RCTs, which ended with negative results. A subsequent case series and an open trial (total n = 21) of another typical antipsychotic drug, haloperidol, were performed, reasoning again that the drive for thinness and fear of weight gain along with a distorted body image is of delusional intensity and poor insight, which may reflect dopaminergic hypoactivity [35,36]. In the 1990s and early 2000s, newer atypical antipsychotics came to the market with a promise of improved efficacy and fewer side effects. Although disillusionment came quickly with severe metabolic side effects noted for many of the drugs, there was promise for AN that these appetite-increasing effects, combined with the antipsychotic’s anxiolytic effect, may prove useful.

Until now, atypical antipsychotics were the most widely studied class of drugs for AN. In total, published clinical trials included almost 400 participants, with approximately 300 of these studying olanzapine. Amisulpride, risperidone and quetiapine [37,38,39] were all tested in a single placebo-controlled RCT, with positive results for weight gain shown for amisulpride (n = 35), which was compared with clomipramine and fluoxetine, but not for risperidone (n = 41) or quetiapine (n = 36), which were compared with placebo.

Drawing on findings that dopamine receptor agonists may positively impact behaviour modification in underweight females experiencing low oestrogen levels and with the aim of investigating weight-neural antipsychotic agents, there has been a growing number of reports of aripiprazole treatment in AN. A case series [40,41,42] and retrospective studies [43] had promising results; however, clinical trials are still lacking.

As mentioned previously and as illustrated in Figure 1, the atypical antipsychotic studied most is olanzapine. The rationale for its use in AN lies in its ability to block dopaminergic and serotonergic receptors, potentially reducing anxiety and obsessions related to food and body concerns while promoting weight gain. This was first observed in non-AN psychiatric conditions and then in early case reports [44]. Four out of five RCTs show olanzapine to be effective in promoting weight gain [32,45,46,47]; however, this effect is small scale. The largest olanzapine RCT, thus far [32], found an increase in body mass index (BMI) of 0.259 over 16 weeks of treatment, compared with 0.095 in the placebo group. While statistically significant, the effect was modest. Additionally, the acceptability of olanzapine in AN treatment was found to be relatively low. Attia et al. reported a high dropout rate of 45% among participants receiving olanzapine [32], which is characteristic of the relatively high dropout rates in the treatment of EDs. These considerations led the WFSBP’s task force [13] to give a Grade 2 recommendation for olanzapine in AN, even though the evidence is strong (Level A). AN is of particular interest in the paediatric population, as the disorder typically presents in adolescents. There were studies in this population, but mixed results for weight gain [48,49,50] prevented efficacy and safety from being established in children and adolescents.

Gastroprokinetic agents are another drug therapy that was proposed for AN, which aims to overcome the delayed gastric emptying that is characteristic of the condition; however, studies on cisapride, metoclopramide and domperidone showed they did not produce any substantial effect [51,52,53]. Appetite stimulants, namely the cannabinoid dronabinol, have shown promising results, but larger studies are needed to accumulate reliable evidence [54].

Over the years, treatment with antidepressants has been investigated from several angles. The comorbidity of AN and depression and the potential for a common pathophysiological mechanism involving catecholamines, as well as weight gain promotion, were behind the first trials in TCAs [55,56] with negative results. SSRIs were subsequently suggested for similar reasons, as well as being an anxiolytic and for anti-obsessional faculties—bearing in mind the high comorbid rates and the potential view of EDs as a disorder on the obsessive–compulsive disorder spectrum [57]. Results for weight gain were mostly negative, while results for other parameters were hard to gauge due to different outcome measures [58,59,60,61]. Addressing depressive symptoms seems to play an important role in the journey to recovery in AN and is considered crucial to the beneficial effect observed in repetitive transcranial magnetic stimulation, for example [62,63]. Most recently, ketamine and esketamine, novel antidepressant treatments, were presented in case reports; these showed beneficial effects in AN, but further studies are needed [64,65].

Attempts to target the hormonal system in AN began early on but, ultimately, are very difficult to implement. Trials involving growth hormones, oxytocin and a ghrelin agonist yielded no significant results [66,67,68]. A recent promising agent in this category is metreleptin, a human recombinant leptin that showed positive results in initial case reports [69,70,71].

## 3. Bulimia Nervosa

The diagnosis of BN was first introduced in 1979 by Gerald Russell’s seminal paper as an “ominous variant AN” [72]. This description was the one that best captured an increasingly notable phenomenon observed in Western societies, leading to its wide acceptance and inclusion in DSM-III and, later, DSM-IV [73]. The criteria were virtually the same as in Russell’s presentation, namely the irresistible urge to overeat followed by self-induced vomiting or purging and a morbid fear of becoming fat. This disorder was considered a new entity unrelated to earlier historical accounts of excessive eating in an indulgent context. The treatment targets defined by Russell were to break the cycle of overeating and vomiting as well as to encourage patients to accept a higher weight [72].

BN came into the picture well into the era of EBM and RCTs. Psychological therapies, the mainstay of BN treatment, were developed and proven to be effective within the frameworks of cognitive behavioural therapy and interpersonal therapy [73]. Alongside these key psychological interventions, pharmacological options were explored.

**Figure 3:**
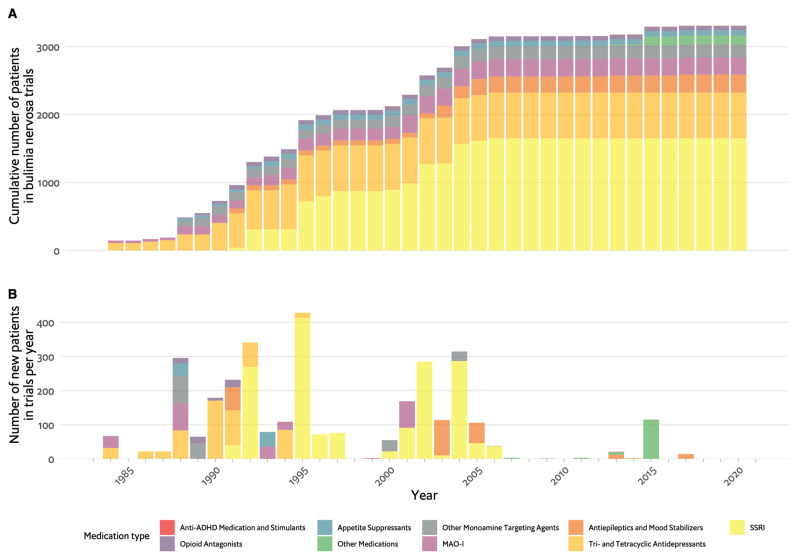
Timeline of pharmacological studies in bulimia nervosa by medication group. (**A**). Cumulative number of patients who participated in studies, colour coded by medication group. (**B**). Number of new participants per year in studies colour coded by medication group. ADHD: attention deficit hyperactivity disorder, MAO-I: monoamine-oxidase inhibitors, SSRIs: selective serotonin reuptake inhibitors.

Figure 3A,B depict the chronological progression of drug therapy studies in BN. Initial studies quickly took the form of RCTs and were published in the early 1980s, soon after the introduction of BN as a disorder. The first drugs examined belonged to the TCA group. These were first introduced in the 1950s when attempts to synthesise drugs that would compete with the success of chlorpromazine yielded imipramine, which demonstrated strong antidepressant effects [74]. The rationale for the use of TCAs in BN was based on their antidepressant action. Dysphoria and depressive symptoms were prominent and common in those with BN, with a high prevalence of affective disorder in their first-degree relatives to the extent that it was proposed to represent a form of affective disorder [75,76,77,78]. Notably, Russell emphasised that antidepressant therapy was appropriate only when depressive symptoms were severe, and such treatment was unlikely to decrease ED symptoms [72]. Pope et al. demonstrated imipramine was associated with a significant decrease in binge eating episodes, decreased preoccupation with food and greater subjective overall improvement [78]. Sabine et al. investigated mianserin and found no significant advantage over placebo in BN symptoms or general psychopathology [79]. These first two studies, with conflicting results, paved the way for further studies of TCAs, 14 in total. Currently, TCAs are no longer recommended due to their side effects and poor acceptability [13].

Monoaminoxidase inhibitors (MAO-Is) were the next group of antidepressant medications to be studied in BN. The case for the use of MAO-Is was their particular efficacy for “atypical depression” and the overlap in symptoms between the depressive syndrome and BN, specifically overeating. Six RCTs produced conflicting results [80,81,82,83,84,85], with phenelzine studied the most and having limited evidence [13]. However, the use of MAO-Is was limited due to the low-tyramine diet required, which complicates the already sensitive nutritional treatment goals [86].

The most widely studied class of medication in BN is SSRIs. Fluoxetine hydrochloride was first described in 1974 as an SSRI, and after 16 years of development and research, it was approved by the FDA for the treatment of depression [87]. This was the earliest of the SSRIs, which have become the most widely prescribed antidepressants and have prescription rates that are continually on the rise, i.e., 1266 per 1000 population in the United Kingdom [88] and 13.2% of American adults reporting antidepressant use in the previous 30 days [89].

The attempt to examine the novel antidepressant fluoxetine arose from the mostly positive results observed in previous trials of antidepressants from different classes, including TCAs, MAO-Is and other monoamine-targeting agents. About 70% of published trials for BN prior to SSRIs had positive results. These trials were limited by small sample sizes, with most trials administering the active drug to under 30 participants, thus considerably restricting statistical power. Actual antidepressant clinical use was further limited by significant side effects, such as the anticholinergic and sedative effects of TCAs and insomnia and blood pressure changes with MAO-Is. This was in addition to the low-tyramine diet required by MAO-Is and weight gain, which was common both in MAO-Is and TCAs [90].

A large-scale multicentre collaborative study was initiated across 13 centres with 387 patients, and fluoxetine at a dose of 60 mg/day was found to be effective for binge/purge reduction [91], leading to its approval by the FDA in 1994 for use in BN. This study was further supported by evidence from four other RCTs [92,93,94,95].

Topiramate is a potent antiepileptic drug that is also widely used for migraine prevention. Following its weight-reducing effects observed in epileptic patients, it was also approved (in combination with phentermine) as an anti-obesity drug [96]. The investigation of topiramate in BN followed preliminary evidence of the effects in BED and case reports. Three RCTs with a total of 172 participants found that topiramate reduced binging and purging in BN, with no significant adverse events or effects reported [97,98,99]. However, an FDA report suggesting that topiramate led to an increased risk of suicide [100], as well as evidence of teratogenic effects and its significant side-effect profile, limited its use. Although it received Grade A evidence, the recommendation for its use is at Level 2 in the recent ED guidelines [13].

Since the last published trial on fluoxetine [101], there have been few substantial drug trials, reflecting the focus on the continuous developments and adaptation of psychological treatments for BN [102]. There were some case reports for aripiprazole [41], an atypical antipsychotic medication also used as a mood stabiliser and antidepressant. Two small-scale open trials of anticonvulsants were reported, one with lamotrigine (n = 14) and the other with zonisamide (n = 12) with positive results [103,104]. The most significant trial was of oxytocin [105], thought to be involved in appetite regulation and to have the potential to reduce binge eating episodes and improve social and emotional processing associated with BN. It was shown that oxytocin reduced 24 h calorie intake in the 34 BN participants compared with healthy controls.

Similar to the pattern observed in AN, the studies in BN mirror psychopharmacological developments in psychiatry over time. The largest studies, with 300–400 participants, were carried out for fluoxetine in the 1990s, at the time SSRIs were being introduced to the market. Figure 2B further illustrates that the majority of trials on antidepressants had positive results, though not all.

## 4. Binge Eating Disorder

Binge eating was first portrayed in 1959 by Albert Stunkard as one of the eating patterns of obese humans [106]. It was described as the consumption of enormous amounts of food in relatively short periods, often indirectly related to stress in a personalised and unconscious manner, which may involve dissociative processes and is followed by discomfort and self-condemnation [107]. It took many years until binge eating was further delineated as a mental disorder or an ED in proposed preliminary criteria tested in field trials [108], and it was finally included in DSM-IV-TR [109] as a research category and with a formal diagnosis in DSM-5 [1]. The initial coupling of binge eating with obesity was, thus, disconnected; obesity, similar to anorexia, is not considered a mental disorder, and while binge eating was found to be more prevalent in people with obesity, it is, nonetheless, found in individuals of normal weight [108].

**Figure 4:**
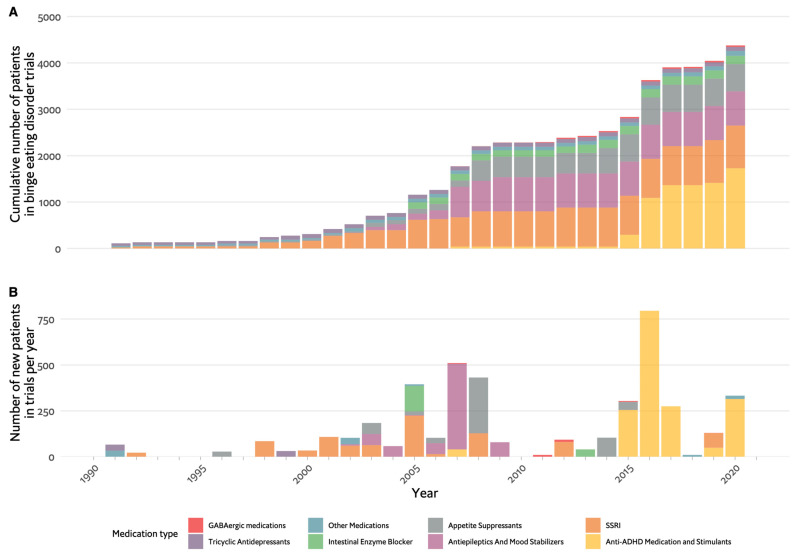
Timeline of pharmacological studies in binge eating disorder by medication group. (**A**). Cumulative number of patients who participated in studies, colour coded by medication group. (**B**). Number of new participants per year in studies, colour coded by medication group. GABA: Gamma-Aminobutyric Acid, SSRIs: selective serotonin reuptake inhibitors, ADHD: attention deficit hyperactivity disorder.

Potential pharmacological treatments were explored before the diagnosis was officially recognised in 2013, which can explain the overlap in the treatment of binge eating with that of obesity. Figure 4A,B show the progression in clinical pharmacologic research with four major medication categories: appetite suppressants, SSRIs, antiepileptics and anti-attention deficit hyperactivity disorder (ADHD) medications. Figure 2C focuses on effectiveness and shows that many types of drugs were found to be effective, but the larger trials focused on the antiepileptic and stimulant categories. The first medication category to be investigated was antidepressants, with few studies of the earlier TCAs initiated in light of their role, albeit partial, in the treatment of BN [110,111,112]. These yielded mixed results with no conclusive recommendations. The use of TCAs as an antidepressant gave way to the more tolerable SSRIs, which were put forward as potential therapeutic agents for BED, again, much because of their role in BN. Fluoxetine, for example, was studied based on its weight-reducing effect observed in depressed and non-depressed populations, as well as its efficacy in BN [113], but only one of four RCTs had positive results [113,114,115,116]. Similarly, other SSRIs, including fluvoxamine, sertraline and citalopram, produced either conflicting or insufficient evidence to produce a sound recommendation [117,118,119,120,121,122].

Appetite suppressants and antiepileptic drugs were next in line in the search for pharmacotherapy for BED. In fact, antiepileptic medications were brought to attention due to the incidental finding of anorexia and weight loss when initially studied in clinical trials in epilepsy and the subsequent superiority to placebo for weight loss in patients with obesity [123,124]. Repeated clinical trials of topiramate, including a large multicentre placebo-controlled RCT, found a consistent reduction in binge eating and reported weight loss [124,125,126], which led to a Grade 1 recommendation for use in the recent WFSBP guidelines [13]. However, as in BN, the dissemination of topiramate as an accepted treatment is compromised by the noted risk for suicidality, contraindication in pregnancy and cognitive side effects [127]. For zonisamide, there was only one positive RCT [128], and for lamotrigine, a single RCT reported weight loss but no reduction in binge eating [129].

The most widely studied appetite suppressant was sibutramine, studied in several RCTs with positive repeated effects on weight loss and reduction in binge eating [130,131,132,133]. However, the drug was ultimately withdrawn in most countries because of its harmful cardiovascular effects [134].

Stimulants and drugs for ADHD have been the focus of drug therapy for BED in the last decade. The reason for turning to anti-ADHD medication was the potential involvement of dopamine (DA) and noradrenaline dysfunction in pathological overeating. Such DA system abnormalities were found in obese individuals, and it was suggested that medications that inhibit the reuptake of DA and noradrenaline could alter pathological binge eating behaviours [135]. Lisdexamfetamine was studied in large-scale placebo-controlled trials and found to be safe and effective, leading to its approval by the FDA as a treatment for BED [135,136,137]. Likewise, dasotraline was investigated in two large-scale (N = 300–400) trials with positive results, but the drug was withdrawn by Sunovion Pharmaceuticals, stating further clinical studies would be needed to obtain regulatory approval for treatment in ADHD and BED, with no specific cause mentioned for the decision [138,139].

## 5. Discussion

This review of the pharmacotherapy of EDs from a historical point of view reveals several important points. First, the development of psychopharmacological treatments for EDs paralleled the advancements in psychiatric pharmacotherapy [28]. As can be seen in Figure 1, Figure 3 and Figure 4, the agents studied either belong to classes of medication studied for other psychiatric conditions, including psychosis, depression and anxiety, or are agents that have a metabolic or hormonal effect, which was hypothesised to be beneficial. No single medication was primarily developed for use in EDs.

**Figure 5:**
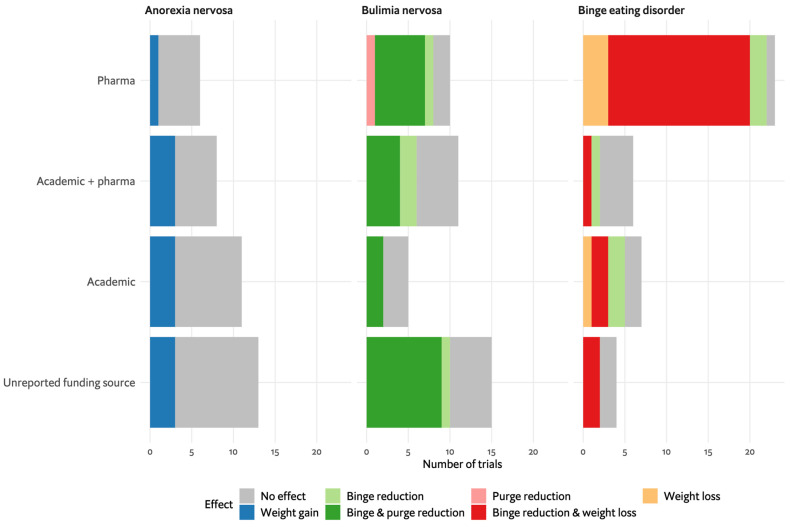
Pharmacological studies according to funding and outcome. Funding was classified as pharma funding if any pharmaceutical involvement was mentioned in the article, whereas specific mention of an academic grant was classified as academic funding. Outcomes include weight gain for anorexia nervosa, binge and purge reduction for bulimia nervosa and binge reduction and weight loss for binge eating disorder or no effects shown.

The application of the RCT model, which is widely used in medicine, including psychiatry and EDs, raises several questions. RCTs were designed for medical conditions with a single causative pathogen, such as bacterial disease, and a clear, isolated outcome [28]. However, EDs are complex, multifactorial disorders [3], and reducing their assessment to a single or a few main outcomes may compromise understanding of a treatment’s effects. In the studies assessed for the WFSBP ED guidelines on which this review is based [13], many trials measured outcomes beyond the key disorder-specific ED symptoms, including depression, anxiety and social skills. However, the lack of uniform tools prevented their overall evaluation and pooling. Data could only be synthesised for core ED symptomatic effects, as is presented in Figure 2 and Figure 5, where effects include weight gain for AN, binge and purge reduction for BN, and binge reduction and weight loss for BED. It appears there is a need to challenge the current RCT approaches in EDs and consider incorporating more dimensional and clinical approaches that capture the complexities of EDs more effectively.

Another significant observation is the relatively small number of participants in ED studies. The majority of studies included 100 participants or less, with 150 participants in the largest AN trial, 400 in the largest BN trial and 750 in the largest BED trial (Figure 1, Figure 2, Figure 3 and Figure 4). The overall number of participants in all ED trials reviewed is roughly 10,000, with fewer than 2000 in AN specifically. This is in contrast to, for example, a meta-analysis including over 100,000 participants for drug treatment in depression [30] or over 50,000 participants in a meta-analysis for pharmacotherapy in schizophrenia [31]. This limited sample size may hinder the generalisability and statistical power of findings. For studies in AN, there is a distinct challenge in recruiting participants. Powers et al. [37], for example, did not make the intended number of participants in their trial of quetiapine, and Spettigue et al. converted a planned RCT for olanzapine to an open trial [44,49,140]. The lack of research funding dedicated to EDs also contributes to the limited number of participants and overall research activity in the field.

The role of funding in clinical pharmacotherapy research is noteworthy. Pharmaceutical industry involvement in pharmacological trials can range from drug supply to the full initiative and execution of studies. In order to raise awareness of potential conflict of interests, and due to concern regarding inappropriate involvement in biomedical research, reporting of funding sources has become obligatory by regulators and standard in papers published in acclaimed journals in past decades [141]. However, the extent of industry involvement may be difficult to accurately gauge as even after journals made such disclosures mandatory in the late 1990s, adherence to uniform requirements has remained poor [142]. Therefore, in the study of EDs, where the number of studies is limited, reviews and guidelines include older studies with unclear pharmaceutical involvement.

In our analysis of AN placebo-controlled studies (Figure 5), only 37% reported any pharmaceutical industry involvement, while 26% of the AN placebo-controlled studies reported weight gain. In the largest and most influential pharmacological study in AN, testing olanzapine with 152 participants, support was partly received by the American National Institute for Mental Health, with Eli Lilly providing olanzapine and matched placebo pills but without further financial support [32]. For BN, 51% of placebo-controlled studies reported pharmaceutical industry involvement, while 63% of the BN placebo-controlled studies analysed reported effects of binge and/or purge reduction. The largest study in BN was conducted on fluoxetine in the early 1990s by the Fluoxetine Bulimia Nervosa Research Group, which was led by researchers from the Lilly Research Laboratories and included up to approx. 400 participants [91,93]. In BED, 72% of placebo-controlled studies reported pharmaceutical industry involvement, with 77% of placebo-controlled BED studies demonstrating drug effects in terms of binge reduction, weight loss or both. For BED, lisdexamfetamine is the most extensively studied medication, with between 50 and 745 participants included in studies sponsored by Shire, which was involved in all aspects of the three larger studies from design to publication [135,136,137,143]. All studies of topiramate [97,99,124,125,144] (but one [98]), which were found to be effective in BN and in BED, were reported to be partly funded by Ortho MacNeil Pharmaceuticals, including assistance in manuscript preparation. Taken together, most of the robust evidence for pharmacological agents in EDs is derived from industry-based studies. ED research is low considering the scope of its burden on patients and society and compared with other mental disorders [8].

Despite the limited evidence for medication efficacy in treating EDs, a considerable proportion of individuals with EDs receive psychopharmacotherapy, which may not be in line with guideline recommendations. Fazeli et al. [145] found that 53% of 524 adults who presented with AN reported using psychotropic medications. Garner et al. [146] reported that 84% of 287 AN patients and 88% of 214 BN patients in specialised treatment facilities were receiving psychotropic medications on admission. Monge et al. [147] observed medication use rates of 20.4% at initial assessment and 59% at 1-year follow-up in adolescent-based ED treatment programmes in the USA. This discrepancy between clinical guidelines and practice was further confirmed by Pardo et al. [148], who looked at medication choice in EDs and observed a low adherence rate (30%) to existing clinical prescribing guidelines, which was associated with better clinical outcomes and lower medication costs.

Several limitations of this review should be noted. First, we only used PubMed as a search engine for the systematic review that formed the basis of the 2023 update to the WFSBP guidelines for the pharmacological treatment of EDs [13]. Although this review is the most comprehensive review of articles on the pharmacological treatment of EDs as yet, the literature search would have been even more thorough if other databases were included, such as Web of Science and PsycInfo, with subsequent removal of duplicates using appropriate software, such as Endnote and Rayyan. In addition, some studies may have been missed in the search process, particularly small and comparably less often cited studies that may not have been picked up by hand searches of reference lists of included papers and related reviews. This is the case for a placebo-controlled trial that tested duloxetine in a subgroup of patients with BED and an additional depressive disorder [149]. Second, an inherent limitation of our historical review is our reliance on published articles. Trial registration databases were not examined to determine which studies were not started, did not manage to recruit, were completed but without evaluation of data or where data were simply not published. Similarly, we assessed for effects of medication and funding based on the published papers themselves. Additionally, we did not investigate the historical circumstances or the motivation of researchers, the scientific trends or the movement of time. Finally, although comorbidities are a major concern in EDs, we did not address this in our review as there is limited research available.

We would also like to mention the current research gap regarding contraindications and medications that should not be used in those with an ED. For example, it is unknown how many patients with AN use topiramate or GLP-1 agonists to lose weight.

## 6. Conclusions

In conclusion, the historical–critical perspective on the pharmacotherapy of EDs highlights the parallel advancements with psychiatric pharmacotherapy. However, the applicability of the RCT model that focuses on a single main outcome, limited participant numbers, inadequate research funding and the need for novel, biologically based treatments call for a re-evaluation of current approaches and a more comprehensive understanding of Eds.

## Data Availability

Not applicable.
